# Silent Aspiration in Guillain–Barré Syndrome Detected by Fiber‐Optic Endoscopic Evaluation of Swallowing (FEES): An Underestimated Risk Factor for Pulmonary Deterioration?

**DOI:** 10.1155/crnm/5947169

**Published:** 2026-11-10

**Authors:** Svetlana Politz Geleva, Sandra Cattenberg, Nicole Rudolph, Ludwig D. Schelosky

**Affiliations:** ^1^ Department of Neurology Cantonal Hospital of Münsterlingen Münsterlingen 8596 Switzerland; ^2^ Clinical Neurology District Hospital Rohrbach Rohrbach 4150 Upper Austria Austria

**Keywords:** case report, dysphagia, fiber-optic endoscopic evaluation of swallowing (FEES), Guillain–Barré syndrome, pneumonia, silent aspiration

## Abstract

Guillain–Barré syndrome (GBS) is an acute, immune‐mediated disorder of the peripheral nervous system that is frequently associated with respiratory complications and bulbar dysfunction. Subclinical dysphagia and silent aspiration represent a significant yet frequently underestimated risk. This paper reports on the course of illness in two patients with particular emphasis on fiber‐optic endoscopic evaluation of swallowing (FEES)–based diagnostics of bulbar dysphagia and the application of the penetration‐aspiration score (PAS). In both cases, silent aspiration of fluids with a PAS score of 8 was initially observed without clinically manifesting respiratory symptoms. While one patient developed aspiration pneumonia eventually, the second patient demonstrated stable respiratory function despite persistent aspiration. The case studies demonstrate that FEES is a highly sensitive method for the early detection of subclinical dysphagia in GBS. Early instrumental swallowing diagnostics have been found to facilitate the detection of aspiration events, the initiation of preventive measures, and the reduction of potential respiratory complications. The prognostic significance of the PAS score with regard to respiratory failure should be systematically investigated in future studies.

## 1. Introduction

Guillain–Barré syndrome (GBS) is characterized by the onset of rapidly progressive quadriparesis within a period of 4 weeks, with or without concomitant cranial nerve palsy, sensory deficit, or autonomic dysfunction, accompanied by albuminocytological dissociation in cerebrospinal fluid [1–3]. Neuromuscular diseases such as GBS are frequently linked to neurogenic swallowing disorders. Facial nerve palsy and dysphagia due to glossopharyngeal or vagal nerve palsy increase the risk of aspiration events. Furthermore, reduced tongue thrust and generalized tongue weakness have been described even at the onset of the disease [4, 5]. These features are often detected late, manifesting as bronchial congestion or aspiration‐induced dyspnea [4]. Approximately one‐third of patients develop respiratory failure during the course of the disease, requiring invasive or noninvasive ventilation [6]. Indicators of the need for mechanical ventilation include rapidly progressive muscle weakness, insufficient coughing, bulbar dysfunction, and a rapid decline in respiratory capacity [3]. These respiratory complications have been identified as significant contributors to morbidity and mortality in GBS [2].

Although GBS patients often require intubation and tracheotomy, the incidence and relevance of neurogenic dysphagia prior to intubation and risk factors for prolonged tube retention remain incompletely elucidated. The objective of this case report was to present fiber‐optic endoscopic evaluation of swallowing (FEES)–based diagnostics of bulbar dysphagia and to analyze the penetration‐aspiration score (PAS) in two GBS patients requiring intensive care.

## 2. Presentation of Case 1

On November 13^th^, 2025, a 59‐year‐old male patient in otherwise good health was admitted to our hospital following a one‐week episode of rhinitis, complicated by sensorimotor disorders of the lower extremities the day before. Neurological examination documented distal paresthesia, accompanied by areflexia of the lower extremities and weakened reflexes of the upper extremities. A predominantly left‐sided proximal arm weakness worsened during the next few days, with the right arm initially elevating above 90° and the left reaching only 50°–60°. At admission, there were slight muscle contractions observable in both legs, but neither leg could be lifted against gravity, while cervical flexion against gravity remained possible. The MRI of the head and spinal cord revealed no pathological findings. The initial lumbar puncture on the next day yielded no remarkable findings; a subsequent puncture on Day 5 after admission, however, exhibited the characteristic albumin‐cytological dissociation, with no serological evidence of other infectious causes (see Table 1; Figure 1). Treatment with intravenous immunoglobulin (IvIg) for 5 days (0.4 g/kg/day) was initiated immediately. The neurography performed 1 week after admission revealed demyelination with prolonged distal motor latency, broadened and fragmented motor action potentials, and absent F‐waves in all leg motor nerves tested (see Table 2; Figure 2). The diagnosis of GBS, most likely acute inflammatory demyelinating polyneuropathy (AIDP), was made [7, 8].

**TABLE 1:** Cerebrospinal fluid profile of Cases 1 and 2.

	Case 1	Case 2	Reference range
Aspect	Clear	Clear	
Color	Transparent	Transparent	
Total protein	1200 mg/L	1240 mg/L	200–500
Total cell count	2/μL	21/μL	< 5

*Note:* CSF, cerebrospinal fluid; mg/L, milligram/L; μl, microliter.

**FIGURE 1:**
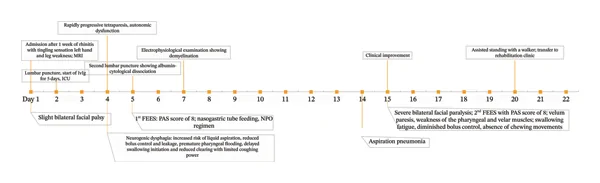
Course of disease of Patient 1. The clinical milestones were displayed above the horizontal axis, whereas the principal swallowing–related events were delineated below it using a distinct framing style. ALT Text: the evolution of Patient’s 1 condition is further delineated graphically, in conjunction with the textual account.

**TABLE 2:** Nerve conduction studies of Cases 1 and 2.

Case 1 (on Day 12)	Distal latency	Action potential amplitude	Conduction velocity	*F* response latency
	ms		m/s	ms
*Median motor left*				
Wrist‐APB	4.26	2.2 mV		32.1
Elbow–wrist	10.3	1.54 mV	41.4	

*Peroneal motor left*				
Ankle	7.66	0.56 mV		Absent
Distal fibula–ankle	21.3	0.22 mV	24.6	

*Tibial motor left*				
Med. malleolus‐AH	8.77	3.0 mV		Absent

*Median sensory left*				
Wrist‐Dig II	5.84	3.2 µV	40.4	NA

*Sural left*				
Calf ‐Med. malleolus	Absent			NA

**Case 2 (on Day 21)**	**Distal latency**	**Action potential amplitude**	**Conduction velocity**	***F* response latency**
	**ms**		**m/s**	**ms**

*Median motor left*				
Wrist‐APB	9.8	3.2 mV		48.1
Elbow–Wrist	16.5	3.0 mV	20.0	

*Ulnar motor left*				
Wrist‐ADM	6.6	5.6 mV		
Dist. sulcus‐wrist	11.9	2.6 mV	41.5	41.0
Prox. sulcus‐Dist. sulcus	15.0	1.9 mV	43.5	

*Peroneal motor left*				
Ankle	12.1	0.2 mV		Absent
Distal fibula–ankle	27.1	0.2 mV	24.6	

*Tibial motor left*				
Med. malleolus‐AH	5.3	0.6 mV		Absent
Knee‐Med. malleolus	26.2	0.1 mV	19.1	

*Median sensory right*				
Wrist‐Dig II	Absent			NA

*Ulnar sensory right*				
Wrist‐Dig V	2.2	2.9 µV	47.7	NA

*Sural left*				
Calf‐Med. malleolus	Absent			NA

EMG M. tib ant.	Fibrillation and PSW abundant, only single action potentials eligible			

*Note:* M. tib ant., tibialis anterior muscle.

Abbreviations: ADM, abductor digiti minimi; AH, abductor hallucis; APB, abductor pollicis brevis; NA, not applicable; PSW, positive sharp wave.

FIGURE 2Nerve conduction studies of Case 1. Median nerve testing showed mildly prolonged distal latency, reduced CMAP amplitude, and minimally slowed conduction velocity, indicating early demyelination with possible mild axonal loss. The common peroneal nerve demonstrated markedly prolonged distal latency, severely reduced CMAP amplitude, profound conduction slowing, and absent F‐waves, consistent with extensive proximal and distal demyelination and probable conduction failure. Tibial nerve findings included prolonged distal latency and absent F‐waves, suggesting additional proximal segment and nerve‐root involvement. Ulnar nerve assessment yielded no further reportable abnormalities. Both cases failed to record sural nerve SNAP. In Case 1, ICU environmental factors precluded acquisition. In Case 2, severe limb edema from paralysis made the procedure technically impossible. Overall, the pattern reflects significant demyelinating dysfunction with superimposed axonal compromise, most pronounced in the lower extremities.

**Figure:**
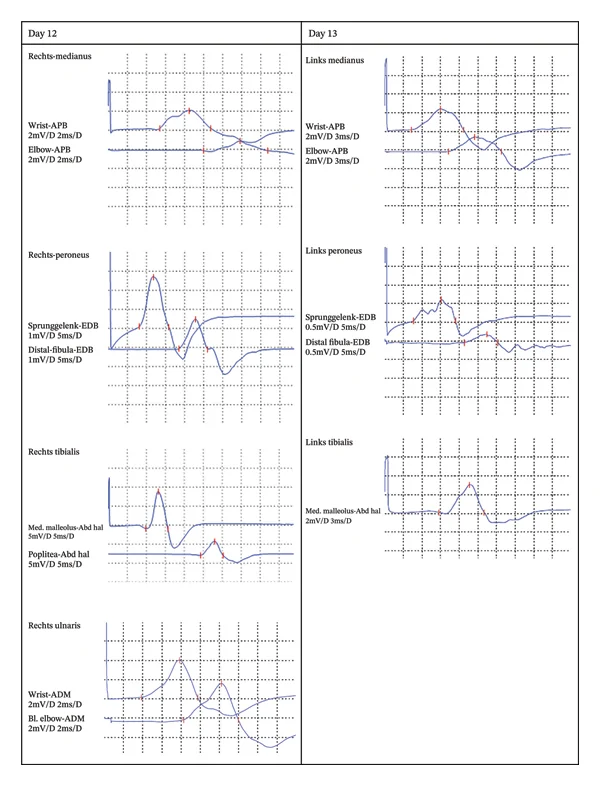

**Figure:**
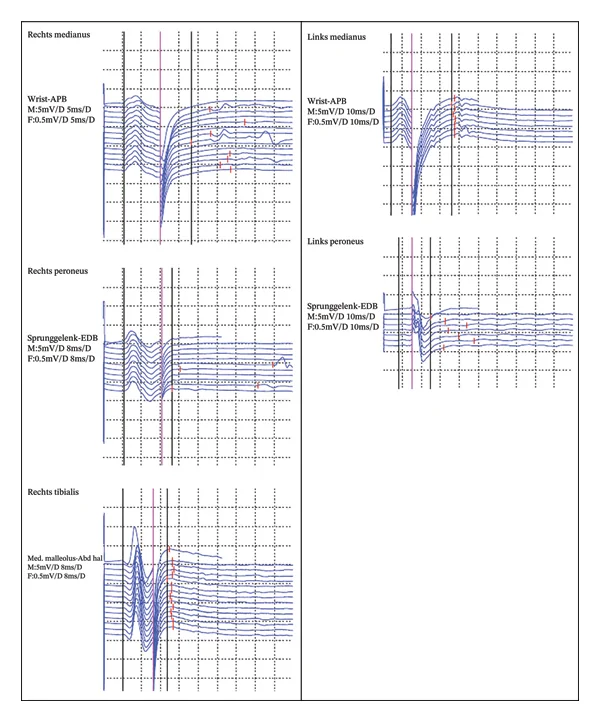

As the disease progressed, the patient developed rapidly progressive tetraparesis, with a focus on the proximal arm muscles and slight bilateral facial palsy. He was transferred to the interdisciplinary intensive care unit (Day 2 after admission). The respiratory muscles remained nearly unaffected throughout the disease course; peak expiratory flow (PEF) values declined only slightly from 480 L/min on Day 4 of the disease course to 410 L/min on Day 6. Overall, the respiratory status remained clinically stable, with peripheral oxygen saturation within the normal range apart from one transient nocturnal decrease to 89%. Serial vital capacity (FVC) or negative inspiratory force (NIF) measurements were not performed. Autonomic dysfunction with extreme hypertensive blood pressure values commenced on the fourth day after admission.

The clinical signs of neurogenic dysphagia became apparent 4 days after admission. A clinical speech and language therapy swallowing assessment revealed facial palsy (Cranial nerve VII), impaired tongue mobility (Cranial nerve XII), and palatal weakness (Cranial nerves IX/X); an increased risk of liquid aspiration with reduced bolus control and leakage; as well as premature pharyngeal flooding, delayed swallowing initiation, and reduced clearing with limited coughing power. The findings of the FEES investigation conducted 5 days after admission corroborated the clinical impression, demonstrating mild sensorimotor dysphagia with silent aspiration of liquids (a PAS score of 8). Based on these findings, nasogastric tube feeding was initiated, and a nil per os (NPO) regimen was started.

Four days later (Day 9 after admission), there occurred a marked clinical deterioration, with the onset of severe bilateral facial paralysis and an inability to close the lips, which prompted one further FEES. Presence of velum paresis and saliva retention in the valleculae and nasopharynx was noted, correlating with the observed weakness of the pharyngeal and velar muscles. The aspiration of liquids (a PAS score of 8) persisted. Following the ingestion of a test bolus with a soft consistency, an exacerbation of swallowing fatigue was observed, accompanied by a concomitant increase in retention in the valleculae and at the upper edge of the epiglottis. This finding is consistent with weak bolus propulsion and difficulty raising the larynx. The clearance was found to be inadequate. Furthermore, the occurrence of bilateral facial nerve palsy resulted in a significantly diminished oral processing phase, accompanied by diminished bolus control and an absence of chewing movements. The paresis led to inadequate bolus preparation and an escalated incidence of leakage.

Thirteen days after admission, diffuse retention and a thrush infection were still present. Eventually, one day later and 9 days after first signs of dysphagia, the patient developed aspiration pneumonia. Pneumonia was diagnosed clinically based on subfebrile temperature, a pulmonary auscultatory focus, leukopenia, increasing CRP levels, yellowish sputum, and worsening oxygenation requiring supplemental oxygen. Microbiological analysis from the sputum sample revealed the presence of *Klebsiella aerogenes, Staphylococcus epidermidis,* and *Candida albicans*. The positive sputum culture was considered supportive for the pneumonia diagnosis rather than definitive evidence, and *Staphylococcus epidermidis* and *Candida albicans,* as part of the normal oropharyngeal flora, may more likely represent colonization rather than causative pathogens. No chest radiograph or CT was performed. Notably, this occurred without any symptoms of dyspnea. An antibiotic therapy with piperacillin/tazobactam was initiated and transitioned to a regimen of cefepime after detection of *K. aerogenes* in the patient’s sputum sample.

Fortunately, the symptoms improved significantly a day later. There was subjective and clinical improvement in regard to the facial nerve palsy, with lip closure now possible again and improved coughing strength. A further FEES was performed. This indicated stable velum function and a secure swallowing pattern without residue formation for mushy food (a PAS score of 1). No secretions were detected in the pharyngeal space. There was also an improvement in the swallowing of liquids, as no penetration or aspiration was detected. However, reduced oral bolus control with pronounced leaking meant that liquids still required compensation. Following the substantial enhancement in findings, the option to resume oral feeding with mushy food was feasible (International Dysphagia Diet Standardization Initiative [IDDSI] Level 4).

During the follow‐up examination 5 days later, a secure swallowing behavior (PAS score of 1) was documented for solid food for the first time. Ingestion of solid foods led to weak propulsion, resulting in residue formation in the valleculae. Soft, mushy, and liquid foods could be swallowed without difficulty, and the retention could be compensated for by swallowing a sip of water. A substantial improvement in the additional neurological impairments was observed, and mobilization was possible up to assisted standing with a walker.

Regular objective dysphagia assessment using FEES was performed to monitor swallowing function and guide individualized swallowing rehabilitation according to the patient’s clinical status and needs.

In summary, mild sensorimotor dysphagia, initially due to involvement of the bulbar cranial nerves, evolved into severe sensorimotor dysphagia, which subsequently subsided during the recovery phase of GBS. This was comprehensively documented and evaluated by FEES, facilitating continuous, patient‐specific dysphagia management with the utilization of assessment parameters including swallowing safety, swallowing efficiency, fatigue, compensatory ability, and the PAS score.

## 3. Presentation of Case 2

The 56‐year‐old female patient, who was otherwise healthy, spent her vacation in Spain. Following a flu‐like infection a few days earlier, the symptoms began on January 21^st^, 2024, with a tingling sensation in the left hand. Later on the same day, her legs gave way while climbing the stairs. The patient was diagnosed with GBS 2 days later, received IvIg (0.4 g/kg/day) for 5 days and was repatriated to our hospital in Switzerland after 20 days (see Figure 3). The subsequent counting of days commences upon admission to our hospital. On admission, she had facial paralysis and difficulty speaking. She was able to lift her arms against slight resistance, but the muscles in her legs showed only minimal contractions without any resulting movement. The other leg functions were paralyzed. Only a mild paresis of the neck musculature was noted, allowing for limited head elevation against gravity. Muscle reflexes could not be elicited in any limb. The patient reported numbness in both hands, lower arms, and legs. Severe autonomic dysfunction with constipation and bladder emptying dysfunction was also present. The patient’s oxygen saturation ranged from 97% to 98%, which was within the normal range.

**FIGURE 3:**
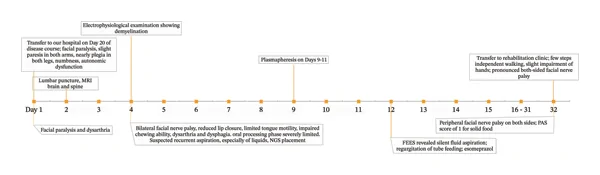
Course of disease of Patient 2. The clinical milestones were displayed above the horizontal axis, whereas the principal swallowing‐related events were delineated below it using a distinct framing style. ALT Text: the evolution of patient 2’s condition is further delineated graphically, in conjunction with the textual account.

Lumbar puncture exhibited albumin‐cytological dissociation (see Table 1), and electrophysiological examination showed neuropathy of all nerves examined, with both severe signs of demyelination and axonal damage (see Table 2). The brain MRI was normal. The spine MRI showed symmetrical caudate fiber swelling and contrast uptake, consistent with GBS, with no evidence of myelitis (see Figure 4). Plasmapheresis was performed on Days 9–11 after admission to our hospital due to the severe and unimproving nature of the disease.

**FIGURE 4:**
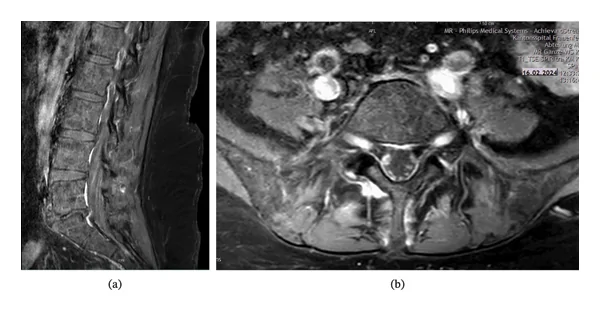
Spinal MRI of Case 2. Sagittal (a) and axial (b) postcontrast T1‐weighted MRI of the lumbar spine demonstrating smooth, symmetric nerve root enhancement consistent with oedematous enlargement of the cauda equina fibres (MR studies courtesy of Dept. of Radiology, Cantonal Hospital Münsterligen, Prof. Dr. Andreisek). ALT Text: Two MRI images of the lumbar spine showing contrast enhancement of the swollen cauda equina fibres.

The initial clinical speech therapy assessment on the fourth day after admission revealed bilateral facial nerve palsy, reduced lip closure, limited tongue motility, impaired chewing ability, dysarthria, and dysphagia. The oral processing phase was severely limited due to the facial palsy. Recurrent aspiration, especially of liquids, was suspected. Dietary assessment was performed using IDDSI Levels 4 and 1. As the patient was unable to meet her nutritional needs orally due to oropharyngeal and sensorimotor limitations, a NGS was placed in addition to partial enteral feeding.

FEES was performed 8 days later, which objectively confirmed the limitations in swallowing identified in the initial clinical examination. Although the clinical speech therapy swallowing examination showed aspiration with intact clearing function, the FEES revealed silent fluid aspiration. IDDSI 4 was maintained, and the level of liquid thickening was increased to IDDSI 2. Furthermore, the presence of regurgitation of tube feeding was revealed through FEES. This finding prompted the administration of esomeprazole 40 mg/d. The objective was to achieve independence from NGS in a relatively short time span.

Despite the regression of bulbar symptoms; recovery of the cranial nerves; and the associated improvement in tongue motility, facial paresis, and dysarthria, the silent aspiration of fluids (a PAS score of 8) persisted. The dietary regimen was adjusted to meet the standards of IDDSI 5, and the thickening of liquids was reduced to IDDSI 1, but silent aspiration persisted until the day of discharge on Day 32 after admission. Consequently, it was recommended that any subsequent dietary adjustments should be made exclusively through the utilization of FEES.

During acute hospitalization, the patient frequently received swallowing rehabilitation customized to her clinical status and needs. She underwent consistent therapeutic taping of the orbicularis oris muscle, which quickly facilitated lip closure and bolus control. Lip and tongue motility were trained by speaking through a cork. Loss of sensitivity in the pharyngo–laryngeal area was compensated for by thickening flavorful drinks that were either very cold or very warm.

32 days after admission and 52 days after onset of the disease, she was transferred to the rehabilitation clinic in a stable condition. She was able to walk a few steps independently again, but the fine motor skills of her hands were still slightly impaired. At the time of transfer, she still had a pronounced peripheral facial nerve palsy on both sides, with slightly incomplete eyelid closure. During the patient’s stay in the acute care facility, no aspiration pneumonia or other respiratory complications were recorded.

The importance of FEES should be particularly emphasized in this case. It was only with its help that it was possible to diagnose, document, and monitor both silent aspiration and regurgitation of tube feedings. Consistent, patient‐specific, low‐risk, and person‐centered dysphagia management was thus made possible, and aspiration pneumonia was prevented.

## 4. Discussion

The cases illustrate important insights into the significance of early FEES examinations in patients with GBS, showing that silent aspiration (PAS score of 8) can occur early on, even when respiratory function is not impaired, and there is no need for ventilation. It suggests that bulbar dysfunction and respiratory insufficiency can have different manifestations and temporal dynamics. FEES detected severe swallowing disorders not evident clinically or in respiratory parameters. A PAS score of 8 is not a direct predictor of respiratory deterioration, but a sensitive marker of subclinical bulbar dysfunction. This bulbar dysfunction may pose a relevant risk of aspiration and secondary pulmonary complications, as demonstrated by the development of aspiration pneumonia in the first case.

The comparison of both courses also suggests clinical heterogeneity: despite identical FEES findings with persistent silent aspiration, one patient remained respiratory and pulmonary stable, while the other patient developed aspiration pneumonia. This finding might indicate that a combination of factors, including but not limited to coughing, secretion management, vigilance, immune status, and the timing of preventive measures, may play a substantial role in the clinical manifestation of the condition. It is important to note that FEES does not provide a prognosis for inevitable respiratory deterioration. However, it does enable the early identification of a vulnerable patient group.

Aspiration pneumonia is a serious complication of GBS, which requires early identification of high‐risk silent laryngeal involvement in dysphagia patients. A number of studies have identified predictors of respiratory failure and invasive ventilation in GBS, including a short interval between symptom onset and hospital admission, advanced disability and cranial nerve involvement, and facial and bulbar weakness. The Erasmus GBS Respiratory Insufficiency Score (EGRIS) integrates these factors into a 0–7 point scale, with higher scores indicating increased risk of respiratory insufficiency. EGRIS allows stratification into low (0–2), moderate (3–4), and high (5–7) risk categories and provides a validated tool for early identification of patients at risk (see Table 3) [3, 5, 9–11].

**TABLE 3:** EGRIS score [3].

Predictor	Categories	Score
Time from onset of weakness to hospital admission, days	> 7	0
	4–7	1
	≤ 3	2

Facial and/or bulbar weakness at hospital admission	Absent	0
	Present	1

MRC sum score at hospital admission	51–60	0
	41–50	1
	31–40	2
	21–30	3
	≤ 20	4

EGRIS total score		0–7

Note: EGRIS = Erasmus Guillain–Barré Syndrome Respiratory Insufficiency Score; risk of respiratory failure/ventilation requirement: score 0–2 = low, 3–4 = moderate, and 5–7 = high.

Abbreviation: MRC = Medical Research Council.

Although cranial nerve involvement and bulbar symptoms significantly influence the clinical course of GBS, the clinical relevance and pathophysiology of neurogenic dysphagia remain insufficiently understood. As previously described by Schröder et al. [12], FEES enables the detection of severe dysphagia and silent aspiration. Our cases complement these observations by demonstrating that FEES can identify clinically unrecognized silent aspiration, while similar FEES findings may nevertheless be associated with different clinical outcomes. Further prospective studies are required to establish evidence‐based indications for the use of FEES in patients with GBS [12]. GBS‐related dysphagia is caused by paresis of the voluntary swallowing muscles (glossopharyngeal, vagal, accessory, and hypoglossal nerves) and by autonomic dysfunction affecting the smooth muscles and reflex mechanisms of the pharynx, larynx, and esophagus. Silent aspiration can result, and repeated or persistent aspiration may lead to alveolar inflammation, ventilation‐perfusion (V/Q) mismatch, hypoxemia and a reduction in respiratory reserve, which increases the need for intubation.

Maskin et al. (2024) found bulbar and cervical muscle weakness independently predicts respiratory failure, and Ogna et al. (2017) highlighted that reduced tongue protrusion force and swallowing–breathing coordination disorders are linked to intubation [11, 13]. The findings of the current study are consistent with those of Orlikowski et al., who showed that tongue weakness correlates more closely with respiratory failure than peripheral paralysis [4]. In a large cohort study, 78% of ventilated patients with GBS developed pneumonia during the course of mechanical ventilation; approximately 59% of all ventilated patients developed early‐onset pneumonia within 5 days after intubation. [14]. This emphasizes that early, often subclinical, bulbar dysfunction may hold greater prognostic significance than motor weakness in the extremities.

In GBS, subclinical dysphagia frequently goes unnoticed. The bedside swallowing test has been criticized for its lack of accuracy. It is supposed that even experienced clinicians miss 40%–50% of patients who are at risk of aspiration during a test [15]. FEES with PAS is a highly sensitive diagnostic procedure for identifying this condition [16]. A PAS score of 8 indicates complete, silent aspiration and an associated elevated risk of complications. However, there is a lack of systematic FEES‐based studies on GBS‐associated swallowing patterns [12, 17]. Previous Video‐Fluoroscopic Swallow Study (VFSS) data from small cohorts have predominantly demonstrated pharyngeal or oral‐pharyngeal dysfunctions, while isolated oral disorders are rare [18]. In the context of pronounced aspiration events in GBS, caused by bulbar symptoms and facial nerve palsy, the question arises as to whether instrumentally obtained swallowing findings (e.g., tongue pressure, swallow‐breath coordination, and FEES with PAS) should be incorporated into risk stratification for respiratory failure. Up until now, the FEES study has had no impact on the EGRIS, and our suggestion is not intended to modify or supplement the EGRIS. However, FEES, as an independent clinical‐apertive examination, can provide additional information about the severity of the tendency to aspirate. This could help identify patients with ongoing aspiration and start preventive measures (e.g., close monitoring, respiratory support, and safe feeding such as IDDSI). Further prospective studies are needed to assess the added predictive value of FEES‐based swallowing parameters for respiratory insufficiency development in GBS patients. Our findings demonstrate that comparable FEES findings may be associated with different clinical outcomes. FEES enables the detection of clinically unrecognized silent aspiration and supports individualized dysphagia management. A PAS score of 8 should not be interpreted as an isolated predictor of aspiration pneumonia, but rather as an indicator of increased aspiration risk with possible associated respiratory complications.

The study is not without limitations. The findings are based on only two cases and therefore cannot be generalized. FEES examinations were performed according to clinical necessity, rather than a standardized protocol. No complementary instrumental diagnostic procedures (e.g, videofluoroscopic swallow study and tongue pressure measurements) were included, and long‐term swallowing outcomes beyond the acute phase were not assessed. Exact duration and intensity of the swallowing rehabilitation interventions were not systematically recorded, as these aspects were not the primary focus of the present investigation. However, the study also demonstrates certain strengths, as illustrated by the early, FEES‐based identification of subclinical dysphagia and silent aspiration in GBS. The repeated administration of instrumental assessments permitted detailed longitudinal monitoring of bulbar dysfunction and swallowing safety. The comparison of two patients with similar clinical characteristics who exhibited divergent respiratory outcomes underscores the heterogeneity of GBS and highlights the potential relevance of FEES‐derived parameters, such as a PAS score of 8, for future risk stratification.

Bulbar dysfunction in GBS should not only be considered in terms of respiratory insufficiency. The present cases suggest that subclinical swallowing disorders with silent aspiration are an early relevant symptom. Timely diagnosis is essential for identifying aspiration events, initiating preventive measures, and avoiding additional respiratory complications. FEES is a sensitive method for detecting this risk, regardless of the current respiratory status. The PAS could serve as an early marker for increased respiratory risk, but requires validation. Future studies may investigate whether pneumonia in patients with GBS is associated primarily with respiratory muscle weakness or with dysphagia‐related aspiration. Pneumonia occurring in the absence of clinically evident respiratory muscle weakness should therefore be considered separately when evaluating respiratory complications in GBS.

## Funding

No funding was received for this study.

## Conflicts of Interest

The authors declare no conflicts of interest.

## Supporting Information

Additional supporting information can be found online in the Supporting Information section.

## Supporting information


**Supporting Information** CARE Silent aspiration in Guillain–Barré syndrome_Politz.

## Data Availability

The data that support the findings of this study are available on request from the corresponding author. The data are not publicly available due to privacy or ethical restrictions.
